# Seeing structure, losing sight: The case for morphological thinking in the age of integration

**DOI:** 10.1002/ar.70011

**Published:** 2025-06-20

**Authors:** Paola Falletta, Erica Tagliatti, Katia Cortese

**Affiliations:** ^1^ Vita‐Salute San Raffaele University, Human Anatomy Milan Italy; ^2^ Experimental Imaging Center, IRCCS Ospedale San Raffaele Milan Italy; ^3^ IRCCS Humanitas Research Hospital, Laboratory of Pharmacology and Brain Pathology Milan Italy; ^4^ DIMES, Department of Experimental Medicine, Human Anatomy, Cellular Electron Microscopy Lab University of Genoa Genoa Italy

**Keywords:** anatomical education, artificial intelligence, electron microscopy, morphology, spatial reasoning, systems biology

In the age of genomics, artificial intelligence (AI), and systems biology, morphology risks becoming an invisible discipline, present in the background, but often unacknowledged in its interpretative power. Biology, however, remains spatial, structured, and shaped. From subcellular compartments to complex organ systems, form continues to inform function. The ability to recognize, describe, and interpret structures across biological scales is a foundational scientific competence. Morphology is not merely descriptive; it is integrative. Morphological sciences bridge developmental, cellular, and evolutionary biology, revealing how gene activity, signaling dynamics, and mechanical forces influence biological architecture. Morphological thinking enables scientists to contextualize molecular processes within the spatial complexity of cells, tissues, and organs. Paradoxically, while technological tools for visualizing structures are expanding rapidly, the number of scientists trained in morphological reasoning is shrinking. If left unaddressed, this erosion threatens to undermine both the scientific depth and translational relevance of biological research. This commentary argues that rediscovering morphology is not a nostalgic act, but a necessary one. We must affirm morphological thinking as a core scientific competence, reframe educational practices to sustain spatial reasoning, and integrate morphology into the evolving world of AI‐driven imaging and systems biology.

Morphology has long been the foundation upon which biological sciences were built. In the 19th century, Georges Cuvier's comparative anatomy laid the groundwork for evolutionary thought by revealing structural homologies. Johann Wolfgang von Goethe's *Metamorphosis of Plants* introduced the idea of structural transformation as a fundamental biological process. Rudolf Virchow's cellular pathology framed disease as a disruption of cellular structure (Byers, 1989). Santiago Ramón y Cajal's neuronal theories, based on meticulous morphological analysis, established the neuron as the basic unit of the nervous system. Morphology was not ancillary to biology's birth; it was its core. Losing morphological reasoning today is not simply a shift in focus; it represents a forgetting of biology's roots. Structural literacy remains essential for understanding the dynamic interplay between form, function, and evolution. Morphological thinking is not limited to cataloging shapes; it is a cognitive skill rooted in spatial reasoning, pattern recognition, and analogical thinking. These cognitive capacities are critical for scientific inquiry across disciplines. Research on spatial cognition, such as the meta‐analysis by Uttal et al. (2013) and the study by Yang et al. (2023), shows that spatial skills are malleable and trainable, and that they predict success in STEM fields. When students engage in dissection, microscopy, or ultrastructural analysis, they are not merely memorizing forms; they are training their ability to manipulate, reconstruct, and interpret complex three‐dimensional relationships. Morphological training develops a “mental toolkit” that extends beyond biology, enhancing the ability to reason about systems, diagnose anomalies, and infer functional implications from structural patterns. This connection between morphology and function was already evident in the 19th century, when microscopy enabled scientists to explore microanatomy in unprecedented detail. One notable innovation of that period was the use of the camera lucida and wax plates to reconstruct microscopic anatomy in three dimensions (Born, 1876, 1883). These reconstructions provided spatial insight still relevant today, as shown by recent work (Maier et al., 2023) and related discussions in the new *The Anatomical Record* special issue on cranial‐based studies (Laitman & Smith, 2025). For a broader historical perspective, Nyhart's *Biology Takes Form* offers an in‐depth analysis of how morphology shaped biological thinking in 19th‐century German universities (Nyhart, 1995).

At the macroscopic level, the decline of dissection‐based education has marked a clear shift in anatomical sciences. Cornwall and Stringer (2021) highlighted how the progressive removal of cadaver‐based dissection from curricula has weakened students' ability to connect anatomical knowledge with clinical and functional relevance. Virtual modeling, 3D visualization, and simulation technologies offer unprecedented educational opportunities, but they also risk flattening the spatial experience of biological form (Estai & Bunt, 2016). Tactile engagement, the physical act of exploring textures, densities, and relationships between structures, fosters embodied spatial cognition that virtual tools cannot fully replicate. Concerns about the decline of structural literacy are not new. Drake et al. (2009) already warned that diminishing structural literacy may compromise both scientific depth and clinical reasoning, emphasizing that hands‐on experiences are critical for the development of integrative thinking in anatomical education. Similarly, Sugand et al. (2010) already emphasized that hands‐on dissection uniquely integrates visual, tactile, and cognitive modalities, enabling students to develop a holistic understanding of anatomy. Morphological thinking is also critical in surgical practice, where the ability to recognize, interpret, and adapt to anatomical variability is fundamental (Carmody et al. (2024)). Surgery is not merely an application of anatomical knowledge, but an active, dynamic interpretation of three‐dimensional biological structures (Older, 2004; Nzenwa et al., 2023). Without morphological reasoning, spatial disorientation and misinterpretation of structural relationships increase the risk of intraoperative complications. More recently, a letter to the editor highlights how digital learning, although valuable, shows limitations in medical education, especially for practical disciplines like anatomy. The authors stress the superiority of face‐to‐face learning for acquiring tactile and spatial skills and call for the integration of hybrid strategies that balance digital and traditional approaches to optimize education in the health sciences (Millán‐Hernández, 2025). Thus, while digital platforms can democratize access to knowledge, they must be deployed as complements, not replacements, to physical engagement. The Anatomical Society's core regional anatomy syllabus (Smith et al., 2016) continues to call attention to the need for direct structural awareness, recognizing that spatial reasoning skills remain fundamental to effective clinical practice.

This detachment extends into microscopic domains. The rise of digital histology platforms has increased accessibility and standardization but also introduces a layer of abstraction. Traditional histological examination through a microscope fosters sensitivity to the real textures, irregularities, and contextual nuances of biological tissues, qualities that are often lost when viewing idealized digital slides. Direct observation of tissues helps the cognitive processes that connect cellular form to function, allowing learners to appreciate biological variability and pathology. Without physical microscopy experience, students and researchers risk becoming detached from the real‐world complexity of biological matter, weakening their ability to recognize meaningful structural variations. At the ultrastructural level, electron microscopy (EM), historically a cornerstone of morphological discovery, is undergoing profound transformation. Modern EM platforms, such as serial block‐face SEM, focused ion beam SEM, and cryo‐EM tomography, enable visualization of biological structures at unprecedented resolution (McCafferty et al., 2024). Correlative light and electron microscopy (CLEM) approaches further bridge the gap between functional imaging and structural analysis (Mäntylä & Verkade, 2024). However, the sophistication and cost of these techniques have driven centralization into large‐scale imaging facilities. While facilities provide technological access, they often separate scientists from their specimens. In many cases, the direct engagement of researchers with the ultrastructural data is replaced by delegation to imaging specialists. This evolution risks reducing morphology to a technical service rather than preserving it as a scientific mode of reasoning. Morphologists must remain active interpreters of EM data, recognizing architectural patterns, structural disruptions, and context‐dependent variations that cannot be captured by molecular signatures alone.

Far from being outdated, morphology is increasingly critical for systems biology. Systems biology aims to model the complex interactions between genes, proteins, cells, and tissues, yet these interactions are inherently spatial and structural. Multiscale models of tissues, such as cardiac conduction systems or tumor microenvironments, require accurate structural maps to simulate physiological dynamics (Burgos‐Panadero et al., 2019; Jorba et al., 2021). Morphological data serve as the ground truth for validating these models. Similarly, emerging fields like spatial transcriptomics and spatial proteomics depend on integrating molecular information within anatomical contexts (Chu et al., 2024). Without structural frameworks, spatial omics risk losing their explanatory power. Morphological reasoning remains indispensable for interpreting complex biological systems across scales. Today, the challenges to morphology are compounded by the rise of AI and machine learning in imaging sciences. AI algorithms have greatly improved at detecting patterns and segmenting structures across datasets of unprecedented size and complexity. In neuroimaging, for example, AI is transforming the detection of subtle morphological changes associated with disease progression (CLAIM, 2024). Recent advances in morphological profiling, enabled by high‐throughput automated imaging and deep learning technologies, are significantly enhancing our ability to understand cellular dynamics and compound mechanisms of action (Tang et al., 2024). However, while AI enhances our capacity to capture and analyze images, it does not replace the interpretative act of understanding form in biological context. AI systems are powerful pattern detectors, but they lack biological insight. Without human morphological reasoning, there is a risk of reducing structural variations and anomalies to mere pixel‐based artifacts, stripped of their biological significance. Beam and Kohane (2018) warned that reliance on AI black‐box models risks undermining clinical and scientific interpretability. Morphologists must evolve to critically engage with AI outputs, serving as arbiters who ensure that imaging data are translated into meaningful biological narratives. Rather than being replaced, the morphologist's role is elevated: becoming a bridge between raw data and biological meaning, especially as imaging techniques span from ultrastructure to whole‐organism scans.

To reclaim and redefine the role of morphology in biomedical science, we propose three strategic directions. First, morphological thinking must be affirmed as a core scientific competence. Morphologists should be recognized not as technicians or illustrators, but as full intellectual partners in multidisciplinary projects. Their insights are essential for imaging interpretation, phenotypic characterization, and the development of biologically faithful computational models. Institutions and funding bodies should formally acknowledge morphology as a core area of expertise. Second, direct engagement with biological structures must be reinforced at all educational levels. From undergraduate education to doctoral training, cadaveric dissection, hands‐on microscopy, and supervised EM training must remain central experiences. Digital innovations should enhance, not replace, physical and visual immersion. Third, morphological expertise must be integrated into AI and imaging pipelines. Rather than viewing AI as a replacement for morphological analysis, imaging systems must be designed with morphological expertise at their core. Morphologists should collaborate with AI developers to ensure that algorithms are trained, validated, and interpreted within a framework of biological relevance.

Rediscovering morphology is not about looking back, but it is a necessary act of scientific clarity. As we build ever more complex representations of biology, from digital twins to organ‐on‐chip models, we must not lose the ability to see and to understand how structure and function are connected. The future of biomedical science will be increasingly visual, integrative, and data driven. In this future, the morphologist must evolve, not into a passive operator of technologies, but into an active interpreter of biological architecture. Morphological reasoning, informed by new tools but rooted in deep understanding, will remain indispensable for bridging the gap between molecular data and biological meaning. To secure this future, we must train a new generation of morphologists: scientists who see, think, and interpret biological form across scales and who recognize that structure and function remain inseparably linked.

## AUTHOR CONTRIBUTIONS

**Paola Falletta:** Conceptualization; writing – review and editing; methodology. **Erica Tagliatti:** Conceptualization; writing – review and editing; methodology. **Katia Cortese:** Conceptualization; writing – original draft; writing – review and editing.

## CONFLICT OF INTEREST STATEMENT

The authors declare no conflicts of interest.
